# School-based health centers: A four year experience, with a focus on reducing student exclusion rates

**DOI:** 10.1186/1750-4732-3-3

**Published:** 2009-03-10

**Authors:** James E Foy, Kathy Hahn

**Affiliations:** 1 Touro University California, College of Osteopathic Medicine, 1310 Johnson Lane, Vallejo, CA 94592-1130, USA; 2 Vallejo City Unified School District, Student Health Services, 665 Walnut Avenue, Vallejo, CA 94592, USA

## Abstract

We describe a four year collaborative experience with an on-site, community school-based health center that is staffed by the Vallejo City Unified School District and supervised by the pediatric faculty of the Touro University College of Osteopathic Medicine, with particular attention to first grade student exclusion rates.

Patient demographics (including payer source), first grade enrollment statistics, and first grade exclusion rates were analyzed using school district enrollment and exclusion data, billing data, and Child Health Disability Program data.

An ethnically diverse patient population is described, with the payer source in 99% of patients being the State of California Child Health Disability Program or no insurance source. Ninety-one percent of office visits were for well child care and immunizations. First grade student exclusion rates for failure to meet the state-mandated physical examination requirement fell 74% over the first four years of the school-based health center's operation.

In summary, our school-based health center serves a patient population that is primarily uninsured. Reduction in first grade student exclusion rates enhances student education and reduces the loss of attendance-based state matching funds. Additionally, our school-based health center has been well accepted by the local community.

## Introduction

The concept of school-based health centers began in the early 1970s in Dallas, Texas and St. Paul, Minnesota, and these centers are now found in elementary schools, middle schools, and high schools, now numbering approximately 1,700 across the nation [1].

However, the concept did not take hold in California until 1987, when a school-based health center was established at San Fernando High School under a Robert Wood Johnson grant. California now has 153 programs state-wide that are providing primary in-school care. Forty-two (27%) are in elementary schools, 14 (10%) in middle schools, 58 (38%) in high schools, 16 (10%) are on mixed-grade campuses, and 23 (15%) are linked, but not on campus, or are in mobile vans [2].

Though they were once controversial, the centers are now viewed as meeting the needs of a population of students that might otherwise go without healthcare, as many children suffer from unrecognized health problems due to lack of access to healthcare. These school-based centers can provide more easily accessible care [3] because they deliver care in a convenient fashion in a familiar and friendly environment that students visit each school day.

School-based health centers meet the needs of pediatric patients without a primary care home, reducing the use of the emergency department for minor conditions [4,5], which unnecessarily taxes our healthcare system and the families themselves. One of the goals of school-based health centers is to reduce these types of visits, and redirect them to the appropriate ambulatory level of care.

In our school-based health center, reducing missed school days is a secondary goal which is beneficial to the student, the working parent, and avoids loss of the school district's attendance-based state funding of approximately $32 per student per day.

## Program history

Efforts to develop the first school-based health center in Solano County began in our local community school district in Vallejo in 2002, with the development of a Parents' Health Advisory Group, to assess health care accessibility. This parent group, meeting with the Vallejo City Unified School District (VCUSD) pediatric nurse practitioner staff and an outreach consultant, reported that traditional medical services were either unaffordable or located too far from home, school and work. Low-income families reported using the emergency department, rather than establishing a medical home.

At that time (2001–2002 school year), rates of documented first grade physicals at the eighteen elementary schools in the VCUSD averaged 61%, with a range of 43% to 91%. These first grade physical examinations are required by the State of California, and if not documented, result in the student's exclusion from school for up to five days. Students may return to school after the five day exclusion, with or without a documented physical examination. However, this exclusion has a negative impact on the child and family, and reduces the school district's state funding. Thus, one of the targeted outcomes for our school-based health center was an increase in the percentage of first grade children receiving a timely physical examination.

Armed with this information, funding sources were approached for grants. Support was received from several sources, and fiscal planning was begun. Pennycook Elementary School was selected as the site, representing a diverse, low-income population with a need. A kitchen attached to the assembly hall at Pennycook was renovated into a small, one examination room medical facility, and in 2004, began to offer services to the children of Vallejo.

Our school-based health center ("the Center") is operated by the VCUSD, with medical supervision provided by the Touro University College of Osteopathic Medicine. The Center was opened in August of 2004 at Pennycook Elementary School in Vallejo, California. The data sources that we analyzed included: 1) billing data, including Child Health and Disability Program (CHDP) billing data; 2) school district attendance data; 3) school district first grade physical exclusion data; and 4) appointment scheduling information.

The Center is open two days a week during the school year, staffed by a certified pediatric nurse practitioner, and supported by one bilingual medical assistant. Services provided at the Center include physical examinations, immunizations, treatment of minor illnesses and injuries, laboratory tests and referrals for dental, optometric and specialty medical services.

The Center serves children between one and eighteen years of age, with the majority being elementary school age. It is open to all children in the community. No patients are refused treatment based on financial considerations.

## Program results

Our patient population ethnicity profile over the three and a half years of operation reveals: 1) 28% Asian; 2) 21% African American; 3) 27% Hispanic; 4) 13% Caucasian and 5) 11% other ethnicity. Billing data reveal that 99% of the patients seen in our Center have no medical insurance, and are financially covered by the CHDP (income criteria less than 200% of the federal poverty level), or have no financial coverage at all.

Our patient encounter data show that 91% of patient encounters were for well child care and immunizations, and 9% were for problem-oriented care. Of course, many of the well child visits also involve care for newly-diagnosed or known chronic medical problems.

California law requires that immunizations be up to date when entering kindergarten, and that upon entry into the first grade the student is required to document a physical examination within the previous eighteen months. Students that do not meet these requirements may be excluded from school for up to five days.

First grade students excluded from school attendance due to lack of a physical examination have dramatically decreased in the years since the Center opened (Table 1). Our school district data base shows that there were 402 first graders excluded in 2004, falling to only 104 in 2007, representing a 74% decrease in first grade student exclusions. School enrollment figures remained relatively static during this four year period (Table 1). Although this decrease may in part be due to a variety of societal factors (fluctuating insurance coverage, increasing parental awareness of pediatric health screening), it is clear that accessibility to the Center played an integral part in the decline of first grade student exclusions.

**Table 1:** Number and percentage of enrolled first grade students excluded from school due to lack of a documented physical examination in comparison to total first grade enrollment per academic year.

**School year**	**Exclusion # (%)**	**Enrollment #**
2004–2005	402 (29)	1407

2005–2006	320 (23)	1369

2006–2007	254 (18)	1382

2007–2008	104 (7)	1390

## Conclusion

Our experience documents a marked decrease (74%) in first grade exclusion rates due to lack of a state-mandated physical examination. These improved rates result in increased school attendance, and directly benefit the school district financially. Additionally, these improved rates have also served to protect the school-based health center from budgetary constraints during times of school district financial difficulties.

Collaboration with community school districts in terms of school-based health center formation and supervision falls within the community service mission of colleges of osteopathic medicine. This collaboration serves the community, and promotes community awareness of osteopathic medicine and its teaching institutions.

School-based health centers are successful because they fill a need. They are located in a convenient, non-traditional setting, where students go on a regular basis. School-based health centers have been shown to provide care in a timely fashion [2], have proven to help children stay in school and improve academic outcomes [6], increase the use of well child services [7], improve immunization rates [7], and reduce the use of expensive emergency room visits [5,7].

National statistics concerning school-based health centers demonstrate that 97% of the patient encounters are for preventive well child care and immunization [8]. This is similar to our experience, with 91% of our patient care visits being for well child and preventive care.

Studies have shown that children without health insurance are four times more likely to go without needed dental care in comparison to children with insurance [9]. Indeed, severe dental decay has also been a great concern in our community, and the children seen in our Center often need dental care and referral.

School-based health centers have been shown to improve asthma care and reduce hospitalizations for childhood asthma [10]. This is particularly pertinent in our community, since our Center is located in Solano County, which has the highest incidence of childhood asthma of all the counties in California [11].

In summary, our Center fills a void that benefits the children, their families and the community that we serve, and augments the safety net system for uninsured and underinsured children. Our experience has documented: 1) medical care provision to a student population that is primarily uninsured; 2) an increased rate of meeting state-mandated first grade physical examination requirements; 3) reduced rates of first grade student exclusion, which improves attendance and enhances student promotion rates [10]; and 4) reduced school district funding losses from state matching funds.

Additionally, our Center has been well-accepted by the local community. Indeed, our efforts have been so well-accepted that we have obtained new grant funding to open a new, larger school-based health center in a second underserved area of Vallejo in the spring of 2009.

## Competing interests

The authors declare that they have no competing interests.

## Authors' contributions

KH provided care to the patients in the manuscript. JF supervised the care to the patients in the manuscript. Demographics, exclusion and enrollment data were obtained from the VCUSD, and summarized by KH. JF conceived of the manuscript. The manuscript was written by JF, with editing assistance by KH. Both JF and KH read and approved the final manuscript.
